# Asymmetrical white matter networks for attending to global versus local features

**DOI:** 10.1016/j.cortex.2015.01.022

**Published:** 2015-11

**Authors:** Magdalena Chechlacz, Dante Mantini, Celine R. Gillebert, Glyn W. Humphreys

**Affiliations:** a Department of Experimental Psychology, University of Oxford, Oxford, UK; b Department of Health Sciences and Technology, ETH Zürich, Zürich, Switzerland

**Keywords:** Figure drawing, Global processing, Local processing, White matter disconnections

## Abstract

The ability to draw objects is a complex process depending on an array of cognitive mechanisms including routines for spatial coding, attention and the processing of both local and global features. Previous studies using both neuropsychological and neuroimaging data have reported hemispheric asymmetries in attending to local versus global features linked to a variety of cortical loci. However, it has not been examined to date whether such asymmetries exist at the level of white matter pathways sub-serving global/local attention. The current study provides a comprehensive analysis of brain-behaviour relationships in the processing of local versus global features based on data from a large cohort of sub-acute stroke patients (*n* = 248) and behavioural measures from a complex figure copy task. The data analysis used newly developed methods for automated delineation of stroke lesions combined with track-wise lesion deficits procedures. We found (i) that reproduction of local features in figure copying was supported by a neural network confined to the left hemisphere, consisting of cortical loci within parietal, occipital and insular lobes and interconnected by the inferior-fronto-occipital fasciculus (IFOF), and (ii) that global feature processing was associated with a right hemisphere network interconnected by the third branch of the superior longitudinal fasciculus and the long segment of the perisylvian network. The data support the argument that asymmetrical white matter disconnections within long–range association pathways predict poor complex figure drawing resulting from deficits in hierarchical representation. We conclude that hemispheric asymmetries in attending to local versus global features exist on the level of both cortical loci and the supporting white matter pathways.

## 1 Introduction

The ability to draw complex visual scenes relies upon on a number of cognitive mechanisms including visual object recognition, routines of spatial coding, feature (global *vs* local) processing, feature binding, attention and planning (for a review see Trojano, Grossi, & Flash, 2009). Our understanding of the role of these different mechanisms in drawing complex objects, as well as the neural substrates supporting the critical cognitive processes, comes largely from neuropsychological studies examining performance on complex figure copy tests (e.g., Chechlacz et al., 2014, Gainotti and Tiacci, 1970, Grossi and Trojano, 1999, Laeng, 2006, Trojano et al., 2009). As visual objects fundamentally consist of hierarchically organized elements, drawing requires attention and representation of multiple levels of object arrangements, including both the overall (global) and local aspects of the scene (Chechlacz et al., 2014, Navon, 1977). Not surprisingly, deficits in the ability to process local and global aspects of form profoundly impact on figure copying performance (Kuschner et al., 2009, McConley et al., 2006, Poreh and Shye, 1998).

Neuropsychological studies in brain-damaged patients as well as functional neuroimaging and transcranial magnetic stimulation (TMS) studies in healthy controls, suggest not only functionally separate brain subsystems but also a striking lateralization of local and global feature processing. These studies provide compelling evidence for right hemisphere dominance for global feature processing and left hemisphere dominance in processing local features of hierarchical visual objects (Delis et al., 1986, Fink et al., 1996, Lamb et al., 1989, Lamb et al., 1990, Mevorach et al., 2005, Robertson and Delis, 1986, Robertson and Lamb, 1991; although see Fink et al., 1997, Marshall and Halligan, 1995, Doricchi and Incoccia, 1998). Specifically, in brain-damaged patients global versus local feature processing deficits have been associated with lesions respectively to the right (global) and left (local) temporo-parietal junction (TPJ) and superior temporal gyrus (STG; Lamb et al., 1989, Lamb et al., 1990). For example by using a simple detection task with letters occurring randomly at either the local or global level, Lamb, Robertson, and Knight (1990) have reported that patients with lesions to the right STG are more prone to global deficits and show a local advantage when identifying hierarchical figures. In contrast, patients with lesions to the left STG are more prone to local deficits and show a global advantage with hierarchical figures. Converging evidence from a study using positron emission tomography has been given by Fink et al. (1996). These authors have found that attention to global aspects of hierarchical forms is associated with activation within the right hemisphere (lingual gyrus), while attention to local features is linked to activation within the left hemisphere (inferior occipital cortex). Furthermore, Mevorach et al. (2005) have shown that TMS applied to temporarily to disrupt the left posterior parietal cortex in right handed-healthy participants results in poor processing of local features (Mevorach et al., 2005). It should be noted that while there is strong evidence suggesting hemispheric specialization, with right hemisphere dominance in global and left hemisphere dominance in local feature processing, some reports advocate that this dichotomy is not universal. For example Fink et al. (1997) conducted a positron emission tomography study in healthy controls and concluded that the involvement of the right hemisphere in global processing, and the left hemisphere in local feature processing, is not universal and strongly depends on the nature of the stimulus used (see also Doricchi and Incoccia, 1998, Marshall and Halligan, 1995).

While most studies agree on some functional lateralization in global versus local feature processing, the implicated cortical loci vary across studies. This too could be potentially explained by variations in the types of visual displays and the tasks used. Alternatively, it may be that the processing of global and local features occurs in several cortical loci, within parietal, temporal and occipital cortices within the right and left hemisphere respectively, interconnected by functionally asymmetrical white matter pathways. To date the white matter substrates of deficits in local versus global feature processing have not been extensively studied. Previous work indicates that interhemispheric interactions facilitating information transfer via the corpus callosum between the left and right hemispheres play a crucial role in global-local processing (Kleinman and Gupta, 2008, Muller-Oehring et al., 2007, Robertson et al., 1993, Robertson and Lamb, 1991, Weissman and Banich, 1999). Besides the few reports suggesting the link between the corpus callosum and hierarchical processing, the overall white matter pathways supporting local versus global processing are poorly understood, and in particular the existence of functional asymmetries within white matter pathways sub-serving hierarchical processing have not been examined.

In the current study we examined brain-behaviour relationships in the processing of local versus global features using data from a large group of sub-acute stroke patients (*n* = 248). Specifically, we aimed to investigate whether functional asymmetries exist on the level of white matter pathways sub-serving global versus local feature processing. Deficits in local versus global processing were measured using the Complex Figure Copy task from the BCoS battery (Chechlacz et al., 2014, Humphreys et al., 2012). In all analyses we treated behavioural measures of performance on the figure copy test as continuous rather than as categorical scores, giving a better opportunity to tease apart the neural substrates of associated cognitive impairments. Furthermore, the approach chosen here did not require us to select patients based on the anatomical characteristics of the lesion; rather we entered into analyses patients who had a wide range of lesions including right as well as left hemisphere cases. This avoided biasing the results based on priori assumptions about patients (e.g., confining the analysis to left hemisphere lesions so that the contribution of right hemisphere lesions is not assessed) and seemed appropriate as previous accounts have indicated lateralized organization of neural networks associated with the processing of hierarchical visual stimuli. As the data were collected as a part of a large clinical trial carried out in the United Kingdom, the analyses were based on computed tomography (CT) scans, which are routinely used in clinical practice. We have previously demonstrated that neuroimaging analyses using CT scans can yield highly reliable and interpretable results (Chechlacz et al., 2014, Chechlacz et al., 2012, Chechlacz et al., 2013, Gillebert et al., 2014). In order to comprehensively examine brain-behaviour relationships we combined here a newly developed toolbox for automated lesion delineation and track-wise lesion-deficit analyses with whole brain statistical analyses using voxel-based morphometry (VBM; Ashburner & Friston, 2000). These procedures allowed us to understand the contribution of both cortical lesions and white matter disconnections to deficits in local and global feature processing.

Our findings demonstrate that asymmetrical organization of brain networks involved in local versus global feature processing occurs both at the level of cortical loci and the associated white matter pathways.

## 2 Methods

### 2.1 Participants

Patients were recruited from several stroke units across the West Midlands (United Kingdom) participating in the multicentre Birmingham University Cognitive Screen project aiming at developing a comprehensive cognitive screen for stroke survivors not contaminated by problems in neglect and aphasia (Humphreys et al., 2012). The behavioural data were only collected from patients who were physically stable, able to understand English, willing to perform the task and had a concentration span of at least 60 min (judged by a multi-disciplinary clinical stroke team). Clinical and demographic data, including CT scans, were obtained from the patients' clinical files. For the purpose of the current study, we excluded patients whose CT scans were unavailable, patients who based on visual inspection of CT scans (by two independent judges) either had enlarged ventricles or poor quality CT scans (in order to prevent artefacts in the neuroimaging analyses). Furthermore, as our analyses were based on newly developed fully automated tool for pre-processing and lesion detection based on CT scans (Gillebert et al., 2014), we have excluded all patients whose scans were not compatible with this method (see below). Finally, we excluded patients who due to severe motor deficits could not complete the figure copy test and lost their ability to use the dominant hand. A total of 248 stroke patients (110 males and 138 females; average age of 72.02 years, range 26–93 years; see Table 1 for full demographic and clinical data) were included. All participants provided written informed consent in agreement with ethics protocols approved by the National Research Ethics Service: Essex 1 Ethics Committee.

### 2.2 Behavioural measures

#### 2.2.1 Complex figure copy test

**Fig. 1:**
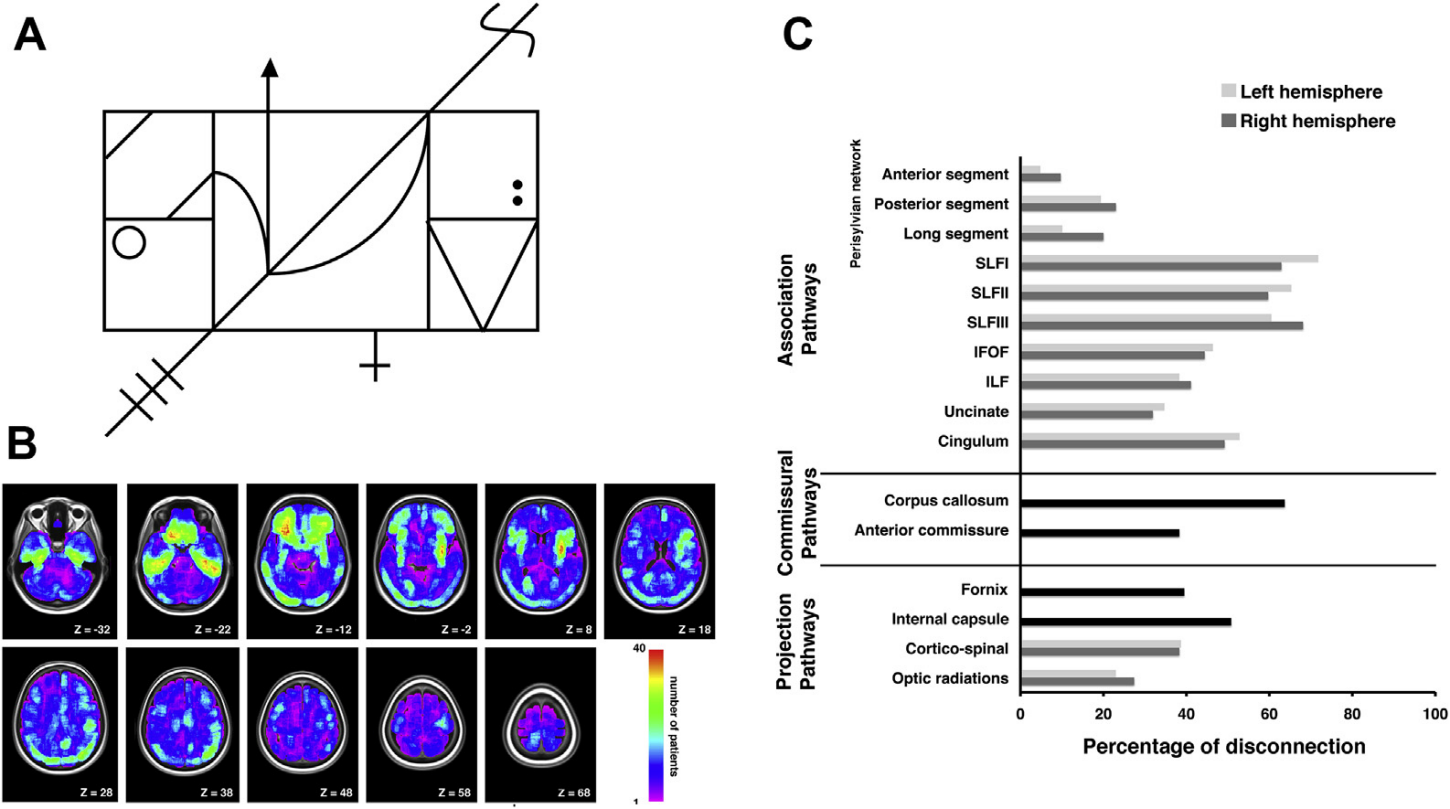
**(A) BCoS Complex Figure Copy task**. Following the instruction, “*“I will show you a figure. Please copy the figure the best you can”,* the complex figure drawing is presented to the patient in the top half of an A4 page. Each patient is given a maximum of 5 min to complete the task. In the original task (Humphreys et al., 2012) performance is scored based on the presence (1 point each), shape/proportion (1 point each) and placement (1 point each) of 5 left elements (diagonal end/3 bars, rectangle, horizontal bar, double oblique bars/parallel and circle), 5 right elements (diagonal end/1 curved line, rectangle, horizontal bar, double oblique/triangle shape and double dot) and 5 middle elements (arrow, right curve, left curve, middle cross and main diagonal line). In addition, the presence and shape/proportion of the middle square is assigned 2 points, thus giving the maximum achievable score of 47 points for the completed task. **(B) Lesion distribution**. Lesion overlap map representing the spatial distribution of lesions among all 248 patients included in the current study. Lesion maps from individual patients were reconstructed using automated toolbox for lesion analyses based on CT scans (Gillebert et al., 2014); see Materials and Methods section for details. The lesion overlap map is shown for nine axial slices in standard MNI space with given MNI Z-coordinates of the presented axial sections. The colour bar shows the number of patients with a lesion within particular voxel. **(C)** Percentage of patients with disconnection in association, commissural and projection white matter pathways within the left and right hemisphere.

The BCoS complex figure consists of a rectangular box divided into 3 global rectangles. Within each of these rectangles are various local features, such as dots, diagonal lines and arrows (see Fig. 1A and Humphreys et al., 2012 for details). Two criteria are used to determine whether patient could be tested on the task: (1) whether the patient was able to hold a pen and (2) whether the patient could make fluent marks/lines on paper. The complex figure was presented to the patient in the top half of an A4 page and the patient was asked to draw an exact copy of the image underneath the original. Patients were given a maximum of 5 min to complete the task. Following the BCoS manual, once the figure has been copied, performance was scored according to whether or not the visual elements were present (1 point each), the correct shapes were represented (1 point each), and whether the elements were assigned their correct position (1 point each), with the maximum achievable score being 47 points (see Fig. 1A for full details of scoring).

The BCoS figure contains a middle square plus two additional rectangles to the left and right with several smaller features inside and thus the scoring of copying performance can be adapted to evaluate different deficits in complex figure copying task. The figure is composed of both global and local features, allowing for the analysis of both global features (e.g., the central square) and local features (e.g., the right double dots) detection deficits. As the present study aimed to examine deficits associated with the processing of local and global features in drawing, we used a modified scoring method to measure these deficits as described below. The modified scoring approach to evaluating performance on the BCoS complex figure test has been previously used and fully described (Chechlacz et al., 2014).

#### 2.2.2 Global/local processing

We measured the reproduction of global and local aspects of the complex figure. Specifically, global features were defined as contributing to the larger layout of the figure, i.e., based on being either larger features and composed of different elements (local features) or having smaller local features located inside them (see Chechlacz et al., 2014). Local features were defined as details that further refined the figure, but were not essential for its identification as a whole, i.e., based on being smaller and simpler elements or part of global features. For this, global features were defined as the larger parts of the figure and included the middle square, the left rectangle, the right rectangle, the left double bar (inside left top square), the right triangle and the long main diagonal line. The local features included: the top left diagonal bar, the parallel bar below it, the left horizontal bar, the left circle, the left diagonal end/three parallel bars, the left curve (inside middle square), the arrow, the right curve (inside middle square), the cross, the right double dot, the right side of the triangle, the left side of the triangle, the right horizontal bar, and the right diagonal end/curved line (“S” shape). Participants were graded according to whether each global or local feature shape was present. The actual shape of the feature and placement of the figure was not taken into account in this analysis. Subjects could score a total of 6 points on the global processing measure and 14 points on the local processing measure. Due to an unequal number of points in assessing global and local processing deficits, the individual patient scores were expressed as a percentage for global and local processing scores and these scores were entered into statistical models used in the lesion-symptom analyses.

### 2.3 Neuroimaging assessment

For all 248 patients included, CT scans were acquired as part of routine clinical assessment following stroke and hospital admission. The average time between the stroke and the CT scan acquisition was 7.63 days (±13.78, with 80% of cases within a week). The neuroimaging data were acquired using the following scanners: Siemens Sensation 16, GE Medical System LightSpeed 16 and LightSpeed Plus. To control for data quality and consistency across different sites, we only used those CT images that were acquired according to specific parameters. Similar CT acquisition protocols (adult head – routine axial scanning) were used across sites. In particular, all the collected CT images covered the whole brain with slices aligned along the AC-PC plane. The in-plane resolution was .5 mm × .5 mm. The slice thickness, however, was not fixed and ranged between 4 and 5 mm. To control for data quality and consistency in our analyses, we excluded CT images with slice thickness larger than 5 mm, as well as those with substantial noise and/or with visible shunts.

### 2.4 Automated delineation of lesions

We adopted a newly developed toolbox for fully automated pre-processing and lesion mapping of brain CT scans (see Gillebert et al., 2014 for full details). The toolbox uses pre-processing procedures implemented in SPM8 (Statistical Parametric Mapping, Welcome Department of Cognitive Neurology, London, UK) and in-house software written in Matlab (The MathWorks, Natick, MA, USA). Subsequent automated lesion delineation for each patient is performed using a voxel-based outlier detection procedure implementing the Crawford–Howell parametric *t*-test for single case–control comparisons (Crawford et al., 2009, Crawford and Howell, 1998). The Crawford–Howell parametric *t*-test is used to generate an outlier t-score map coding the degree of abnormality of each voxel compared to the normal range based on control CT scans. Next, the t-score maps are thresholded to generate binary lesion maps in MNI space (Gillebert et al., 2014). The lesion map in MNI space is also converted to the native CT space allowing the researcher to verify lesion reconstruction based on the original CT scan. As all further analyses in the current paper were based on automatically generated lesion maps, we have excluded those scans were quality of the image was visibly poor or the results of the automated lesion detection were poor due to image quality or lack of visible lesion on the scan.

The binary lesion maps for all patients were used in the track-wise lesion-deficit analyses and to calculate lesion volumes. The lesion volume for each patient was calculated using Matlab (The MathWorks, Natick, MA, USA). Finally, to provide complete evaluation of the neuroanatomical correlates of global versus local processing, we have supplemented the track-wise lesion-deficit analyses with whole brain statistical analyses using voxel-based morphometry (VBM; Ashburner & Friston, 2000).

### 2.5 Track-wise lesion-deficit analyses

To assess the relationship between white matter damage and global/local processing deficits we performed track-wise lesion deficit analyses based on an approach (Thiebaut de Schotten et al., 2014) utilizing diffusion tensor imaging (DTI) tractography atlases of human white matter tracts (Thiebaut de Schotten et al., 2011a, Thiebaut de Schotten et al., 2011b). By using the patients' reconstructed lesion maps (in MNI space), and the maps of white matter tracts from the above atlases (also in MNI space), we first evaluated the pattern of disconnection within white matter tracts (association, projection and commissural) for each individual patient. Our analyses were based on an atlas map of association pathways (inferior longitudinal fasciculus, inferior-fronto-occipital fasciculus (IFOF), perisylvian network: anterior, posterior and long segment, cingulum, uncinate, superior longitudinal fasciculus segments I, II and III), commissural pathways (anterior commissure and corpus callosum) and projection pathways (fornix, internal capsule, optic radiations and cortico-spinal tract). All maps of white matter tracts represent a probability of a given voxel belonging to that tract and these maps were overlapped with the patients' lesion maps. We next calculated a continuous measure of the pathway disconnection by calculating the size of the overlap (in cubic centimetres) between each patient's lesion map and each thresholded (50%) pathway map using Matlab (The MathWorks, Natick, MA, USA; Thiebaut de Schotten et al., 2014). This threshold has been chosen based on data indicating age related changes (age related decreases in the size of the reconstructed white matter pathways; Stadlbauer, Salomonowitz, Strunk, Hammen, & Ganslandt, 2008) and individual variability in peripheral sections of the tracts (Thiebaut de Schotten, Ffytche, et al., 2011). This approach allowed us to consider only the anatomical core of each tract and not the variable periphery (Thiebaut de Schotten et al., 2011b, Thiebaut de Schotten et al., 2014). We used these continuous measures of white matter disconnections in the statistical track-wise lesion-deficit analyses based on linear regression. In the linear regression we entered the lesion volume, age and each individual pathway disconnection measure as independent variables to test whether the disconnection within specific pathways (controlling for lesion volume and age) predicted global or local processing deficits (using continuous behavioural measures with scores expressed as a percentage for global and local processing). We also performed additional regression analysis including two extra independent variables indicating visual problems within left and right visual field based on measure of unilateral misses (within left and right visual field) from the BCoS visual extinction test (Humphreys et al., 2012).^1^ The regression analyses were carried out separately for the left and right hemispheres of each patient. Each track-wise lesion deficit analysis was subjected to Bonferroni correction for multiple comparisons (α level; *p* = .003 based on 16 tracts analysed). SPSS 21 software was used (IBM SPSS Statistics, NY, USA) to compute linear regressions in order to identify, which white matter pathways when damaged predicted the presence of visual search deficits.

### 2.6 VBM

All the CT scans were pre-processed using SPM8 (Statistical Parametric Mapping, Welcome Department of Cognitive Neurology, London UK) as previously described (Chechlacz et al., 2012, Chechlacz et al., 2013). Briefly, the images were first re-aligned manually along the anterior-posterior-commissural (ac-pc) axes and then normalized (Ashburner & Friston, 2003) to an in-house CT template. Next we used the unified segmentation algorithm as implemented in SPM8 (Ashburner & Friston, 2005) with six standard tissue class priors indicating the probability of finding expected signal sources of grey matter (GM), white matter (WM), cerebrospinal fluid (CSF), fat, bone and air (i.e., six different tissues classes), at each voxel of the image. As the CT scans were acquired following stroke, to account for the presence of any abnormal tissue, we also included an additional, seventh tissue class corresponding to the lesioned tissue according to a previously described modified segmentation protocol (Seghier, Ramlackhansingh, Crinion, Leff, & Price, 2008). The probability for an abnormal voxel (lesioned voxel) lying within the grey and white matter was estimated based on the ratio between the average lesion size and the grey plus white matter voxels (Chechlacz et al., 2012, Chechlacz et al., 2013). The segmented GM and WM images were smoothed with a 12-mm FWHM Gaussian filter to accommodate the assumption of random field theory used in the statistical analysis (Worsley, 2003).

In order to compute correlations between the behavioural measures of deficits in global and local feature processing and GM lesions we employed random effects analyses within the general linear model framework (Ashburner and Friston, 2000, Kiebel and Holmes, 2003). We used the full factorial design to generate a single model including as main covariates global and local processing scores (expressed as a percentage); to control for potential confounding factors we also used as covariates age, gender, handedness and lesion volume. We only report results that showed significant effects at *p* < .001 cluster-level corrected for multiple comparison with the amplitude of voxels surviving of *p* < .001 uncorrected across the whole brain and an extent threshold of 800 mm^3^ (>100 voxels). The brain coordinates are presented in standardized MNI space. The anatomical localization of the lesion sites associated with constructional apraxia was based on the Anatomical Automatic Labeling toolbox (AAL toolbox, Tzourio-Mazoyer et al., 2002), the Duvernoy Human Brain Atlas (Duvernoy, Cabanis, & Vannson, 1991) and the Woolsey Brain Atlas (Woolsey, Hanaway, & Gado, 2008).

## 3 Results

**Fig. 2:**
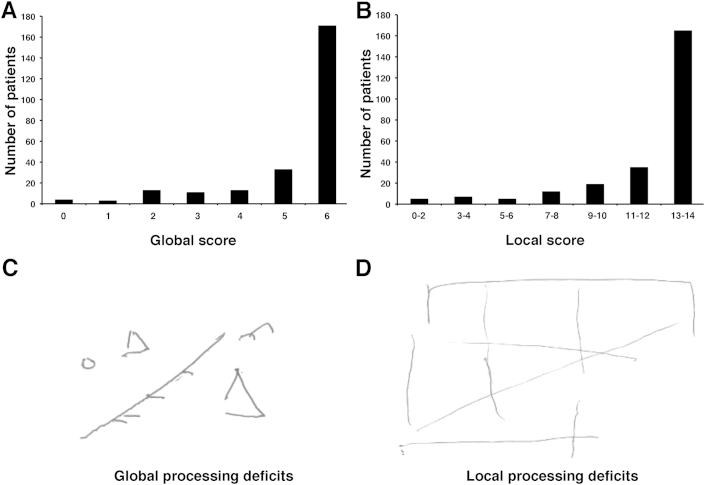
Distribution of **(A)** global and **(B)** local processing scores in the performance on the BCoS Complex Figure Copy Test in the studied group of patients (*n* = 248). Examples of patients' performance on BCoS Complex Figure Copy consistent with diagnosis of **(C)** global and **(D)** local processing deficits.

The overall lesion distribution within both hemispheres is illustrated in Fig. 1B and the percentages of patients with disconnections in (i) association, (ii) commissural and (iii) projection white matter pathways within the left and right hemispheres are shown in Fig. 1C. We subsequently examined the neural correlates of deficits in global versus local processing as assessed by performance on the BCoS complex figure copying test. Fig. 2 shows the distribution of global and local scores in the studied group of patients as well as examples of the BCoS complex figure test performance illustrating global and local processing impairments. We found no difference in performances between male and female patients [global processing, t(246) = 1.14, *p* = .25; local processing, t(246) = .26, *p* = .80].

### 3.1 Hodological track-wise lesion-deficit analyses

**Fig. 3:**
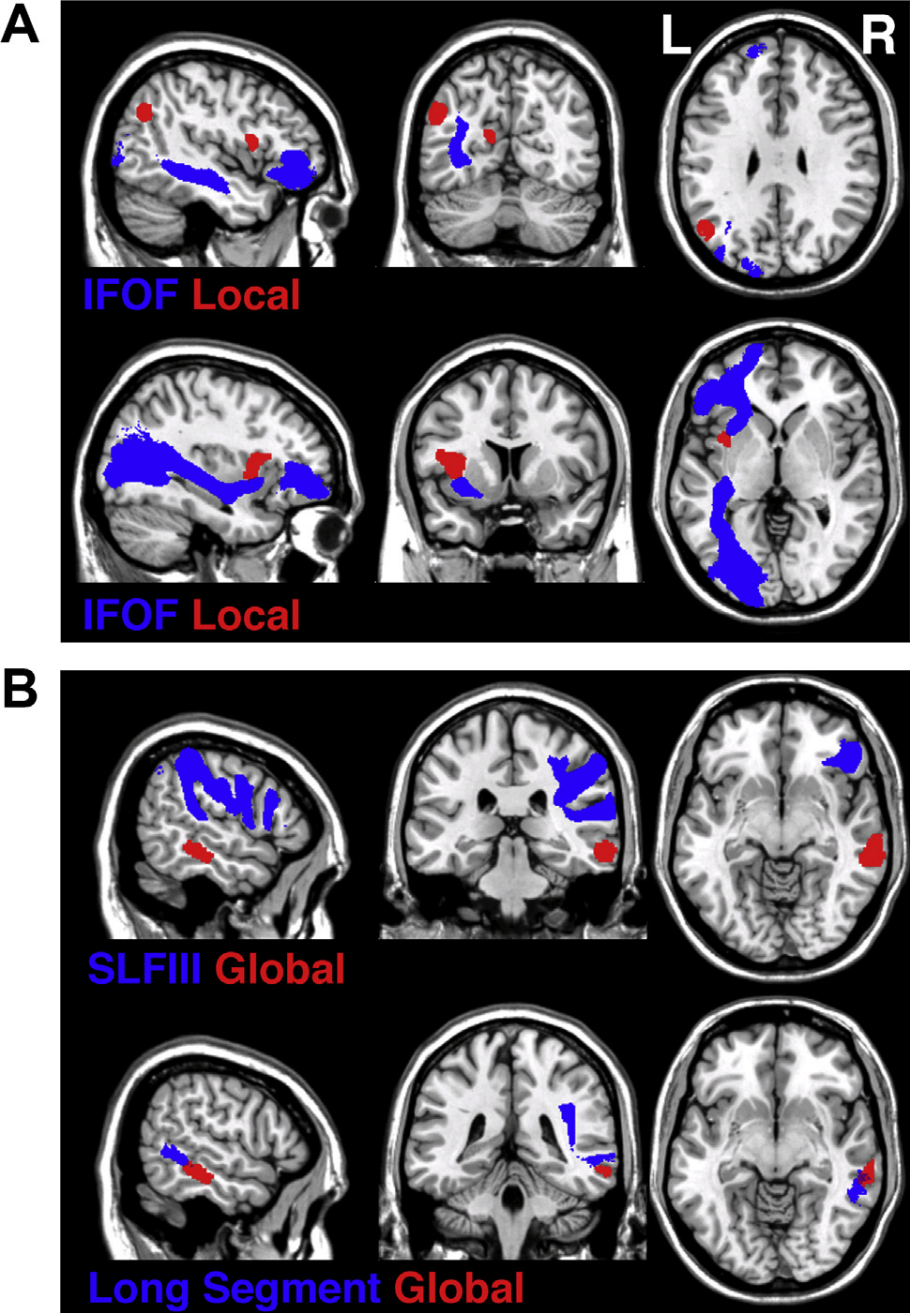
The asymmetrical brain networks for attending to **(A)** local and **(B)** global features. The trajectories of white matter pathways (blue) indicated by track-wise lesion analyses are presented in relation to cortical loci indentified by VBM (red). The trajectories of white matter pathways (blue; IFOF = inferior-fronto-occipital fasciculus, SLFIII = third branch of superior longitudinal fasciculus and long segment of perisylvian network) are presented as the thresholded (50%) maps from the DTI tractography atlas of human white matter tracts (Thiebaut de Schotten et al., 2011a, Thiebaut de Schotten et al., 2011b) and the cortical loci (red) are presented as binary statistical maps thresholded at the significance level of *p* < .001 cluster-level corrected for multiple comparison (see Table 2 for full details).

We adopted here a hodological approach to understanding the contribution of white matter disconnections to cognitive symptoms (Catani and Mesulam, 2008a, Catani and Mesulam, 2008b, Rudrauf et al., 2008, Thiebaut de Schotten et al., 2008) based on linear regression performed to identify specific white matter pathways, which when damaged predict deficits in local versus global processing in complex figure copying. The linear regression analysis indicated that lateralized damage within the left and right hemispheres contributes specifically to local versus global processing deficits respectively. Damage within the left IFOF was a predictor of local processing deficits in the complex figure copy test (*β* = .210; *p* = .001; Fig. 3A), while damage within the right SLFIII (*β* = .213; *p* = .001; Fig. 3B) and the long segment of perisylvian network (long segment of arcuate fasciculus; *β* = .189; *p* = .003; Fig. 3B) were predictors of global processing deficits.

As a control analysis, we repeated the regression analysis additionally controlling for the presence of left and right visual field deficits. The analysis confirmed the left IFOF as a predictor of local processing deficits in the complex figure copy test (*β* = .182; *p* = .002) and damage within the right SLFIII (*β* = .217; *p* = .0001) and the long segment of perisylvian network (long segment of arcuate fasciculus; *β* = .196; *p* = .0001) as predictors of global processing deficits.

We failed to observe any reliable results for the link between disconnections within left hemisphere pathways and global processing deficits or any reliable results for the link between disconnection within right hemisphere pathways and local processing deficits. Thus, our hodological analyses clearly indicated that asymmetrical white matter disconnections predict deficits in local versus global feature processing.

### 3.2 Topological VBM

Alongside the track-wise lesion deficit analyses we used VBM to examine GM substrates of global versus local processing deficits. The VBM data presented here were based on a subset of the patients reported in Chechlacz et al. (2014) and we repeated the VBM analysis here to supplement the data concerning the white matter disconnection. The current VBM results matched our previous findings. Fig. 3 illustrates the asymmetrical brain networks for responding to local and global features i.e., the trajectory of white matter pathways indicated by track-wise lesion analyses in relation to cortical loci indentified by VBM. The deficits in local feature processing were linked to left hemisphere damage within the inferior parietal lobule (mainly angular gyrus), the insula and the calcarine cortex extending into the cuneus and precuneus (see Table 2 and Fig. 3A), while deficits in global feature processing were linked to right hemisphere lesions involving the middle temporal gyrus (MTG) extending into the inferior temporal gyrus (ITG; Table 2 and Fig. 3B).

## 4 Discussion

The current study investigated (i) the white matter pathways sub-serving local versus global processing for reproducing a complex drawing and (ii) whether hemispheric asymmetries in feature processing are determined solely by lateralized cortical functions or whether are supported by functionally lateralized white matter pathways. Our combined hodological and topological analyses indicated that deficits in hierarchical representation in figure copying were indeed linked not only to lateralized damage within cortical loci but also to asymmetrical white matter disconnections within long–range association pathways. It is increasingly clear that many cognitive processes depend on the function of large neural networks comprising multiple cortical areas. Consequently, cognitive deficits may arise not only from damage to discrete cortical loci but also from damage to either different remote cortical areas forming specific functional networks or damage within white matter pathways interconnecting different remote cortical loci (Catani and Mesulam, 2008a, Catani and Mesulam, 2008b, Catani and Mesulam, 2008b, Rudrauf et al., 2008, Thiebaut de Schotten et al., 2008, Thiebaut de Schotten et al., 2014). The findings presented here support the notion that hierarchical processing depends on function of large-scale neuronal networks, and that damage to specific white matter pathways can predict deficits in the production of local and global properties of complex figures.

We demonstrated that the reproduction of local features in figure copying was supported by a neural network confined to the left hemisphere, consisting of cortical loci within parietal, occipital and insular lobes interconnected by the IFOF. These findings support previous studies linking the left hemisphere damage to impaired local feature processing (see for example Chechlacz et al., 2014, Fink et al., 1996, Lamb et al., 1989, Lamb et al., 1990). Specifically, our VBM analysis highlighted the role of the left inferior parietal lobule (mainly the angular gyrus), the left insular cortex and the left calcarine cortex extending into the cuneus and precuneus. In addition to the left parietal and occipital cortices, which have previously been functionally linked to local feature processing (e.g., Chechlacz et al., 2014, Fink et al., 1996, Mevorach et al., 2005), earlier studies have also indicated critical loci within the left temporal cortex (left STG; Lamb et al., 1989, Lamb et al., 1990). Taken together the data suggest a widespread network sub-serving local feature processing within the left hemisphere. The link between the insula and attention to local features was somewhat surprising as, on the whole, the insular cortex has been linked to auditory, sensorimotor, pain, taste and emotional processing (for recent review see Chang et al., 2013, Jones et al., 2010). Some previous studies indicated that lesions within insula contribute to visuospatial attention deficits associated with poor drawing and copying in neglect and constructional apraxia patients, but these findings has been reported following right and not left hemisphere lesions (e.g., Chechlacz et al., 2010, Corbetta and Shulman, 2011, Russell et al., 2010). Interestingly, recent studies combining meta-analyses approaches with connectivity-based parcellation indicate that the insula is an exceptionally diverse structure and should be considered as a central functional hub in several interconnecting networks in the human brain (e.g., Chang et al., 2013, Uddin et al., 2014). In terms of serving as a processing hub, the insula may be important for integrating the perceptual and attentional information necessary for reproducing the local parts of complex figure. Here such integration may require both attention to the local elements and the identification of those elements, and the insula may be important for this integration process.

Interestingly, our track-wise lesion deficit analyses linked the impairments in local feature processing to damage within the left IFOF, a long association pathway interconnecting ventral occipital cortex with the frontal lobes (Catani et al., 2002, Catani and Thiebaut de Schotten, 2008, Thiebaut de Schotten et al., 2012). Post-mortem anatomical dissection studies indicate that the IFOF consists of at least two subcomponents, one connecting frontal and temporal structures and the other connecting frontal, parietal and occipital structures and that some deep IFOF fibres enter the insular cortex (Martino et al., 2010, Sarubbo et al., 2013). Thus we propose that the IFOF provides a critical link between multiple cortical loci (within the parietal, insular, temporal and occipital lobes) that support the processing and reproduction of local forms. Our findings also extend the growing body of evidence linking the IFOF to visuospatial attention, visual processing and selection (Aralasmak et al., 2006, Doricchi et al., 2008, Fox et al., 2008, Rudrauf et al., 2008, Schmahmann et al., 2007, Schmahmann et al., 2008). However, a note of caution should be added here. While post-mortem dissections provide evidence of a relationship between IFOF projections and insular regions (Martino et al., 2010, Sarubbo et al., 2013), there is no direct evidence that the IFOF carries axons originating from the insula. Finally, while our current work and previous studies link the IFOF to visuospatial processing, there is a large body of evidence linking the left IFOF in particular to language networks, notably in semantic processing (e.g., Almairac et al., 2014, Axer et al., 2013, Dick and Tremblay, 2012, Duffau, 2008, Khan et al., 2014). The support for the role of left IFOF in language processing, extending the classical focus on arcuate fasciculus in language functions, is based on studies using diverse methodological approaches including lesion-symptom mapping, electrostimulation and tractography (Almairac et al., 2014, Axer et al., 2013, Catani et al., 2005, Catani and Mesulam, 2008a, Dick and Tremblay, 2012, Duffau, 2008, Khan et al., 2014). However, it is difficult to attribute the present deficits in reproducing local aspects of an abstract (non-nameable) pattern with language impairments, and there seems no a priori reason why language impairments should impact on local rather than more global aspects of form. We suggest instead that the left IFOF can serve more than one cognitive function. Taking into account that the IFOF is considered the longest associative pathway in the human brain extending from frontal lobes via temporo-basal and parietal areas into occipital cortex (Catani et al., 2002; Catani & Thiebaut de Schotten, 2008; Thiebaut de Schotten et al., 2012), this is perhaps not surprising.

In contrast to the data on local feature processing, we demonstrated that processing and reproducing global features in complex figures is supported by the right hemisphere. Our data indicated that global feature processing errors were associated with damage to the right MTG extending into the ITG. This finding is in agreement with previous work linking right temporal cortex to global feature processing (see Doyon and Milner, 1991, Lamb et al., 1990), however previous studies examining global feature processing have indicated mainly the TPJ and the STG (Lamb et al., 1989, Lamb et al., 1990). One potential explanation of these disparities is that the studies varied in the nature of the visual displays and the tasks assigned to participants. Alternatively, and similar to the argument we have made concerning local feature processing, the processing of global form may rely on a widespread neuronal network incorporating several cortical regions. The latter argument is supported by our data demonstrating that disconnections within two long association pathways – the SLFIII and the long segment of perisylvian network within the right hemisphere predict deficits in global feature processing. Interestingly, both pathways provide connections to frontal areas, not traditionally associated with global feature processing, but which are nevertheless part of the fronto-parietal attention network. The SLFIII connects inferior parietal lobule with the inferior frontal gyrus and functionally has been implicated in linking regions within the ventral attentional network underlying capture of spatial attention by salient targets (Corbetta et al., 2000, Corbetta and Shulman, 2002, Thiebaut de Schotten et al., 2011a, Thiebaut de Schotten et al., 2012). The long segment of the perisylvian network (the long segment of the arcuate fasciculus) connects areas within the frontal and temporal lobes (Catani et al., 2002, Catani et al., 2005, Thiebaut de Schotten et al., 2012) and within the right hemisphere this pathway has been functionally linked to visuospatial processing mainly based on data from neglect patients (Doricchi et al., 2008, Thiebaut de Schotten et al., 2008). Our findings here extend the known functionality of these two pathways, linking them to the processing and representation of global features.

While the results presented in the current study are in line with numerous reports advocating that there is asymmetrical organization of brain networks involved in local versus global feature processing (Delis et al., 1986, Fink et al., 1996, Lamb et al., 1989, Lamb et al., 1990, Mevorach et al., 2005, Robertson and Delis, 1986, Robertson and Lamb, 1991), there are also some contradictory accounts. Notably some findings indicate that the involvement of the right hemisphere in global processing, and the left hemisphere in local feature processing, is not universal and strongly depends on stimulus category. For instance, in a study using positron emission tomography in healthy participants Fink et al. (1997) observed greater right hemisphere activation for local features and greater left hemisphere activation for global features processing in task using object-based hierarchical stimuli. We note however that this is the opposite pattern to a prior report by the same group (Fink et al., 1996) with letter-based hierarchical stimuli. Furthermore, there is neuropsychological evidence from brain injured patients suggesting that the attentional abilities to perceive local versus global features cannot be simply explained by the proposed hemispheric specialization and the functional interplay between two hemispheres plays an important role (Doricchi and Incoccia, 1998, Marshall and Halligan, 1995, Robertson et al., 1993).

In relation to these findings, one caveat of the current study should be considered. While most of the previously published work has been conducted using compound stimuli in which smaller, independent local features (e.g., small letters; Navon test; Navon, 1977) make up a single larger, global object (e.g., a large letter), our report is based on a test requiring the copying of a complex figure made up of local elements link to the more global structure. It is possible that the extra demands on segmentation here, to represent the local figures for sequential copying, may have also led to hemisphere specialisation – for example, recruiting right hemisphere process not to represent global information but rather to segment the global structure from the local elements (see Straube, Grimsen, & Fahle, 2010). Hence we should be cautious in proposing exactly which processes are affected.

While our findings support the notion that hemispheric specialization in attending to local versus global features exists not only on the level of cortical loci but also the sub-serving white matter pathways, we have not found evidence in line with previous reports indicating the role of interhemispheric connectivity via the corpus callosum. To date several studies not only suggest that communication between left and right hemisphere via the corpus callosum is critical for global-local processing but also provide evidence that these processes are significantly affected following commissurotomy or microstructural changes within the corpus callosum (Kleinman and Gupta, 2008, Muller-Oehring et al., 2007; Muller-Oehring et al., 2010; Robertson and Lamb, 1991, Robertson et al., 1993, Weissman and Banich, 1999). The results of our study in no way contradict previous findings. It is plausible that the discrepancy between our and earlier studies result from differences in methodological approaches such as the studied patient populations and test instrument used. A point of caution should also be raised in relation to the track-wise lesion deficit analyses based on an approach utilizing tractography derived white matter atlases (Thiebaut de Schotten et al., 2014, Thiebaut de Schotten et al., 2011a, Thiebaut de Schotten et al., 2011b). Atlas maps based on DTI tractography might suffer from underestimation of white matter fibres within regions of crossing and fanning fibres. This results from the simple fact that tensor model fails to fully describe the complexity of white matter architecture in such regions (Basser, Pajevic, Pierpaoli, Duda, & Aldroubi, 2000). These shortcomings would particularly affect callosal and cortico-spinal fibres. However, please note that the SLF maps (SLF I–III) used here were reconstructed based on spherical deconvolution tractography, a diffusion technique less affected by crossing fibres (Thiebaut de Schotten, Dell'Acqua, et al., 2011).

## Figures and Tables

**Table 1:** Patient details: clinical and demographic data (*n* = 248, all stroke patients included in the current study).

	Mean value or number of patients	Std. deviation
Age in years	72.03	13.13
Sex (male/female)	110/138	N/A
Handedness (Right/Left)	229/19	N/A
Aetiology (ISCH/BL)	224/24	N/A
Lesion volume (cubic centimetres)	21.94	32.04
Stroke -CT scan in days^a^	7.63	13.78
Stroke-BCoS in days^a^	20.83	17.12
**BCoS Complex Figure Copy Test**		
Complex Figure – full score	34.25 (47)^b^	11.06
Local feature processing score	12.0 (14)^b^	3.09
Global feature processing score	5.26 (6)^b^	1.39

a Interval between stroke onset and CT scan or cognitive assessment based on BCoS.

b Mean score across the entire group of patients, the number in brackets indicates maximum score for a given test; BL, bleed (hemorrhagic stroke); ISCH, ischaemic stroke; N/A, not applicable.

**Table 2:** Grey matter substrates of local and global processing deficits.

Model	Cluster level		Voxel level	Coordinates			Brain structure (location)
P_FWE_	Size	Z-score	X	Y	Z
**Local feature processing**							
	.000	926	3.36	**−40**	**11**	**12**	Left insula
.000	481	3.33	**−50**	**−63**	**33**	Left IPL (angular gyrus)
.001	143	3.20	**−14**	**−66**	**16**	Left calcarine extending into cuneus and precuneus
**Global feature processing**							
	.000	884	5.18	**69**	**−30**	**−12**	Right MTG

Abbreviations: IPL, inferior parietal lobule; MTG, middle temporal gyrus.

## Footnotes

1 Testing for visual extinction was done by the examiner raising his/her left and right index fingers on either side of his/her head and then moving (two brief bending movements) either left or right (unilateral trials) or both fingers simultaneously (bilateral trials). The task consisted of 4 unilateral left, 4 unilateral right and 8 bilateral trials (Humphreys et al., 2012).
